# Clinical application of transcranial magnetic stimulation for functional bowel disease

**DOI:** 10.3389/fmed.2023.1213067

**Published:** 2023-06-16

**Authors:** Guangyao Li, Binghui Jin, Zhe Fan

**Affiliations:** ^1^Department of General Surgery, The Third People’s Hospital of Dalian, Dalian Medical University, Dalian, China; ^2^Department of Central Laboratory, The Third People’s Hospital of Dalian, Dalian Medical University, Dalian, China; ^3^Liaoning Province Key Laboratory of Corneal and Ocular Surface Diseases Research, The Third People’s Hospital of Dalian, Dalian Medical University, Dalian, China

**Keywords:** functional bowel disorder, brain-gut axis, brain imaging, transcranial magnetic stimulation, treatment

## Abstract

Functional bowel disorder (FBD) is a common gastrointestinal disease syndrome characterized by dysmotility and secretion without known organic lesions. The pathogenesis of FBD is still unclear. In recent years, with the rise of neurogastroenterology, it has initially revealed its close relationship with the “brain-gut axis.” Transcranial magnetic stimulation (TMS) is a technique for detecting and treating the nervous system, that is characterized by non-invasiveness and painlessness. TMS plays an important role in the diagnosis and treatment of diseases, and provides a new method for the treatment of FBD. In this paper, we summarized and analyzed the research progress of using TMS therapy applied to patients with irritable bowel syndrome and functional constipation by domestic and foreign scholars in recent years by means of literature search, and found that TMS therapy could improve the intestinal discomfort and accompanying mental symptoms in patients with FBD.

## 1. Introduction

Functional bowel disorder (FBD) is one of the common diseases of the digestive system, which is characterized by intestinal sensory, secretion, and motor dysfunction, and is a group of intestinal diseases with recurrent episodes without organic lesions (1). The pathogenesis of FBD is not fully understood and is thought to be the result of complex interactions between physiology, psychology, social influences, genetic susceptibility, and early life experiences (2). In recent years, with the development of neurogastroenterology, researchers have conducted in-depth studies of the “brain-gut axis” using brain imaging techniques and have confirmed the influence of central brain activity on the pathophysiological changes in FBD (3). The activity of the cerebral cortex affects the motility of the gastrointestinal tract through afferent and efferent pathways and is regulated by nerves. As a non-invasive, painless, safe, and reliable technique, TMS has achieved remarkable clinical results, and has now become one of the hot spots in the treatment of FBD (4). This article reviews the classification of FBD, brain imaging of FBD under different stimulation conditions, and the effect of TMS on FBD.

## 2. Functional bowel disease and classification

FBD is one of the most common diseases in the clinical practice of gastroenterology, accounting for more than 40% of the patients visiting the gastroenterology department (5). FBD refers to a disease in which symptoms such as bloating, abdominal pain, diarrhea, and constipation appear in the digestive system, but without organic lesions. In the 2016 release of *Rome IV: Functional Gastrointestinal Disorders*, FBD includes six disorders: irritable bowel syndrome (IBS), functional constipation, functional diarrhea, bloating/abdominal distention, nonspecific functional enteropathy, and opioid-induced constipation (OIC) (6, 7). Rome IV revised the diagnosis of the first five diseases, with opioid-induced constipation as a new disease. From the diagnostic criteria established by the Rome Commission, we can see that the clinical manifestations of the various subtypes of functional enteropathy are a combination of one or more symptoms, i.e., the symptoms of each type can be converted to each other and there are obvious repetitions (8). In view of this, the experts of the Rome IV Committee proposed a conceptual framework for FBD, namely that FBD has the same pathophysiological mechanisms and that different diseases can be seen as a continuous spectrum of diseases with varying frequency, intensity, and severity of symptoms (9).

## 3. “Brain-gut axis” and brain imaging of functional bowel disease

With the publication of the Rome IV, the “brain-gut axis” is considered to be the basis of the pathophysiological changes in FBD, and there is increasing evidence to support that the evolution of functional enteropathy occurs in three stages: stage 1—gastrointestinal motility disorder; stage 2—visceral hypersensitivity; and stage 3—bidirectional brain-gut axis dysfunction (3, 10, 11). The brain-gut axis is a bidirectional regulatory axis linking the brain to the gastrointestinal tract through a neuro-immune-endocrine network (12). The central, autonomic, and enteric nervous systems are the main pathways of the brain-gut axis (13). In this model perspective, emotions, thoughts, and perceptions affect gastrointestinal sensation, secretion, motility, immune regulation, mucosal inflammation, and permeability (14). Conversely, altered or disturbed gastrointestinal function can also affect conscious perception and behavior in the brain (2). This bidirectional signal can lead to dysregulation of the autonomic nervous system, which may play an important role in the pathophysiology of irritable bowel syndrome (15). However, it is not feasible to directly assess brain-gut axis function in humans. Therefore, researchers are currently focusing on brain stimulation and brain imaging techniques to better understand the cortical functions associated with intestinal function and functional dyspepsia (16). Recent advances in functional brain imaging techniques and their availability as research tools in clinical studies have facilitated the study of FBD, especially regarding the role of the central nervous system and the brain-gut axis in their pathophysiology. There is new evidence that the central nervous system in patients with IBS may process noxious visceral stimuli abnormally. Changes in brain structure and function are observed on magnetic resonance imaging both before and after repetitive transcranial magnetic stimulation (rTMS) (17). Although the results varied, they all involved the anterior cingulate cortex and prefrontal cortical areas (18). Increased regional cerebral blood flow in the anterior cingulate cortex and insular cortex activates the hippocampus, thalamus, and hypothalamus in response to visceral stimulation (19). The frequency and pattern of rTMS also change the resting state activity of neurons at the stimulation site and adjacent sites, and the magnitude and pattern of changes in neuronal activity depend on the frequency and pattern of rTMS (20). It follows that the use of brain stimulation and brain imaging can provide a better understanding of the relationship between gut motility and the cerebral cortex. By looking at the results presented by brain imaging and then using brain stimulation to modulate the movement of the gut, the researchers have provided a theoretical basis for TMS to treat FBD.

## 4. Transcranial magnetic stimulation and its effect on functional bowel disease

### 4.1. TMS in the medical field

The stimulation methods of brain neurons are mainly divided into electrical stimulation and magnetic stimulation (21). The electrical stimulation of brain neurons acts on the cerebral cortex with a weak polarized direct current and affects the membrane potential of the nerve cells under the electrode site by the action of an electric field, causing depolarization or hyperpolarization of the resting membrane potential. The altered polarization of the membrane is the main mechanism for the different effects following electrical stimulation. However, the operation of direct current stimulation is cumbersome and traumatic, which will cause obvious discomfort to the patient. The existence of the skull will lead to a large attenuation of the stimulation current. Therefore, it is relatively difficult to use direct current stimulation to stimulate deep tissues. Compared to electrical stimulation, TMS is a non-invasive brain stimulation technique that generates a short, rapidly changing magnetic field to induce an electric current in the brain, which has a strong penetrating ability in the brain and fundamentally solves the problem that electrical stimulation cannot access deep stimulation (22). In general, single-pulse and double-pulse TMS are used to explore brain function, while rTMS is used to induce changes in brain activity that can persist beyond the stimulation period (23). The excitability of the stimulated area, as well as the intensity and frequency of the stimulation, determines the changes in brain activity.

Functional magnetic resonance imaging (fMRI) studies have shown that the effects of rTMS induce changes in distal brain regions, with low frequency (<1 HZ) rTMS producing inhibitory effects and high frequency (>5 HZ) rTMS producing excitatory effects (24). The persistent effects after rTMS are not only manifested in motor cortical areas but also in occipital, prefrontal, parietal, and cerebellar sites, a phenomenon that makes it possible to apply rTMS to modulate excitability and inhibition in the corresponding cortical areas (25). Although TMS has only been in development for a few decades, it has demonstrated great therapeutic potential in several clinical areas. It has also shown great possibilities in the study of brain function. In terms of diagnostic, TMS is less costly and more reliable, while in terms of therapeutic, TMS is safer, more convenient, and more acceptable to patients than electrical stimulation. TMS has been widely used in medical treatment for epilepsy (26), Parkinson’s (27), depression (28), neuropathic pain (29), and stroke (30), but there are few studies on TMS applied to FBD, which is also a relatively novel treatment.

TMS has been studied in patients and healthy volunteers around the world for more than 25 years, and the results support the safety and reliability of TMS by evaluating its adverse effects through meta-analysis (31). The most common side effect of TMS is headache or neck pain, which is usually transient and either relieves spontaneously or with over-the-counter pain medication. A rare and serious potential adverse effect of TMS is seizure induction, but fewer than 20 cases of TMS-induced seizures have been reported in recent years, with an induction rate of less than 1 in 1,000. In individuals susceptible to epilepsy, the risk is higher, but the incidence of triggering remains relatively low, at about 1%–2% (32). In general, the risk of serious adverse reactions is low as long as safety guidelines and recommendations are followed. Rosa summarized and analyzed thousands of cases treated with rTMS, only 6 cases had transient seizures. The adverse effects are usually closely related to the extent of the patient’s condition, the concomitant underlying disease, tolerance, and frequency of stimulation. The side effects are minimal and not statistically significant compared to the clinical outcome, so rTMS is considered a safe and non-invasive technique (33).

### 4.2. The therapeutic effect of TMS on functional bowel disease

Although the Rome Committee classified FBD into 6 different subtypes, the clinical manifestations of different classifications have similar and repetitive phenomena, and may have the same pathophysiology (5). Constipation, abdominal pain, diarrhea, abdominal discomfort, and anxiety, depression, and mental distress caused by recurrent episodes of FBD are the main symptoms that plague patients (34). TMS has only been used in the treatment of FBD for just over a decade but has yielded exciting results for patients with FBD (22). Because research on TMS applied to FBD is just beginning, researchers at home and abroad have mainly investigated the improvement effect of TMS on constipation and IBS. In the current study, researchers found that TMS significantly improved bowel movements in patients with functional constipation and also significantly improved anxiety and depression associated with the chronic condition (35). The main clinical manifestation of IBS patients is abdominal pain, which is also significantly improved after TMS treatment (36).

#### 4.2.1. TMS improves functional constipation

Functional constipation is one of the common types of FBD. Recurrent episodes of persistent constipation have a huge impact on the physical and mental health of patients, and over time, most patients experience anxiety and depression. Chronic anxiety and depression can affect the hypothalamus, plant nerves and inhibit peripheral autonomic nerves, triggering aggravation of constipation and thus increasing the burden of treatment. TMS therapy not only improves the patient’s constipation symptoms but also significantly reduces the patient’s mental state, which is beneficial to the recovery of the disease (37). Patients with functional constipation using TMS of motor evoked potentials (MEP) showed a significant decrease in MEP wave amplitude (reduced number of firing neurons) and a general increase in latency (slowed nerve conduction velocity). Impaired conduction of the cortico-lumbosacral-anorectal nerve pathway in patients with functional constipation may be responsible for the significant reduction in MEP wave amplitude. Scholars have applied fMRI and PET studies to reveal that the occurrence of these disorders is associated with abnormalities in the central cortical or subcortical pathways of the brain. TMS motor evoked potential (TMS-MEP) assessed the status of the cortico-spinal-anorectal motor nerve efferent pathway, providing information on the efferent pathway of the brain-gut axis (38).

In the clinical efficacy observation of patients with functional constipation treated with TMS combined with electroacupuncture, Li et al. (35) found that after 4 weeks of TMS treatment, the severity (CAS score decreased from 7.31 ± 2.66 to 4.08 ± 1.04), frequency (SBM number increased from 2.62 ± 0.65 to 5.15 ± 1.14, CSBM number increased from 1.31 ± 0.85 to 3.31 ± 1.44), stool character (BSS score increased from 2.92 ± 1.26 to 4.08 ± 0.76) and mental status (SDS score decreased from 58.62 ± 8.53 to 42.77 ± 4.15 and SAS score decreased from 56.46 ± 4.88 to 37.08 ± 6.50) were improved. After using paroxetine combined with low-frequency TMS to treat depression with functional gastrointestinal disease for 4 weeks, Jiang et al. found that the Hamilton depression scale (HAMD) score in the control group decreased from 21.7 ± 4.0 to 15.3 ± 4.1 in 34 cases, the Hamilton anxiety scale (HAMA) score decreased from 17.5 ± 3.8 to 12.9 ± 4.4, the constipation history impression scale (CGI-S) score decreased from 3.7 ± 0.8 to 2.9 ± 0.6. Only a small amount of dizziness and headache or blood pressure fluctuations (±≥10 mmHg) occurred in both groups during treatment, and the incidence of side effects was not statistically significant (39). The use of TMS in patients with functional gastrointestinal disorders may facilitate the recovery of the patient’s disease and mental status. Cheng et al. (40) used TMS to treat patients with functional gastrointestinal diseases for 4 weeks and found that the levels of neuropeptides (94.82 ± 18.53 to 118.53 ± 22.29) and substance P (35.16 ± 8.42 to 47.65 ± 11.06) in the treatment group were significantly improved.

TMS treatment effectively improves gastrointestinal motility, mediates emotions, and relieves symptoms by regulating the brain-gut axis, providing a safe, non-invasive, and effective treatment for the clinical treatment of functional gastrointestinal diseases. Long-term constipation will have a negative impact on the patient’s mental condition. More than half of the patients with constipation are accompanied by severe anxiety or depression. In the long term, in a state of high tension, anxiety can cause brain and intestinal axis dysfunction, which in turn aggravates the constipation condition, forming a vicious circle. TMS not only regulates the brain-gut axis disorder in patients with functional constipation but also significantly improves the patient’s anxiety and depression, which is more conducive to the treatment of constipation (35).

#### 4.2.2. Effectiveness of TMS for irritable bowel syndrome

The Rome Commission has identified abdominal pain as a necessary condition for the diagnosis of IBS, and abdominal pain is often the main cause of distress in IBS patients (34). Although the pathogenesis of IBS is not cleared, at least half of the patients with IBS have visceral hypersensitivity, which is associated with abnormal processing of visceral injurious signals within the central nervous system. Research confirms that IBS is associated with changes in structural brain activity involved in pain processing (41). The application of repetitive low-frequency TMS increases the rectal pain threshold in patients with IBS, thereby reducing their abdominal pain symptoms. TMS applied to the motor cortex also induces analgesic effects in patients with chronic pain syndrome (42). Several neuroimaging studies have shown that TMS in the motor cortex causes not only local changes in brain activity but also bilateral changes in some distal cortical and subcortical areas, including some involved in pain processing. The analgesic effect of rTMS may be caused mainly by alterations in the central pain modulation system (43).

In Melchior’s experiment, it was found that while rTMS had only a slight improvement in pain threshold (VAS score decreased from 3.8 ± 2.2 to 3.0 ± 2.3), maximum tolerance and rectal compliance in IBS patients compared to healthy controls, in IBS patients with rectal hypersensitivity (pressure pain threshold ≤ for 40 mmHg), rTMS significantly increased rectal volume pain threshold and decreased pain threshold (VAS score decreased from 4.0 ± 2.4 to 3.2 ± 2.2). All patients showed a significant improvement in the sensation of abdominal pain. The failure to achieve the expected results may be related to the small sample size (only 16 patients), the different staging of rTMS, and the fact that most rTMS patients have a history of taking painkillers [painkillers can reduce rectal sensitivity (44)] (36). However, the therapeutic effect of rTMS in patients with rectal hypersensitivity is exciting. Algladi et al. (45) found that high-frequency cortical rTMS could modulate experimentally induced anorectal pain after using TMS in IBS patients. Anal pain thresholds were increased immediately, 30 and 60 min after application of 10 Hz rTMS, but anal sphincter sensation was barely affected. Similarly, increased rectal pain thresholds were found immediately, 30, and 60 min after the use of 10 Hz rTMS, with the difference that rectal sensory thresholds were increased 60 min after the use of 10 Hz rTMS. Somatic effects are enhanced with increasing duration and intensity of rTMS. Activation of the motor cortex may play a role in modulating affective factors of pain, and downward stimulation of the brainstem would block pain transmission. Li et al. (46) found that stimulation of the dorsolateral prefrontal cortex (DLPFC) significantly improved pain, mood, and quality of life in patients with refractory IBS with depression after using rTMS. This may be due to stimulation of the dorsolateral prefrontal cortex resulting in altered metabolism in the distribution area.

IBS is a chronic pain condition, and the use of active pain management and placebo therapy is not effective. Pain-relieving therapy is mostly treated with opioids, which will cause adverse reactions such as constipation over time. The placebo may have a biological analgesic response, causing adverse effects that may be more pronounced than the pain relief. TMS has been preliminarily shown to be effective for pain relief in IBS and may provide a safe, effective, and well-tolerated alternative therapy for IBS patients in the near future instead of conventional drugs (see Table 1).

**Table 1:** Transcranial magnetic stimulation (TMS) improves the clinical symptoms of FBD.

References	Objects	Methods	Results
Li et al. (35)	Patients with functional constipation	Transcranial magnetic stimulation combined with electroacupuncture treatment	Improvement in severity of constipation, frequency of bowel movements, stool characteristics, and mental status
Jiang et al. (39)	Patients with functional gastrointestinal disease and depression	Paroxetine combined with low frequency transcranial magnetic stimulation	Constipation and mental status improved
Cheng et al. (40)	Patients with functional gastrointestinal disorders	Transcranial magnetic stimulation	Elevated levels of neuropeptides and substance P
Melchior et al. (36)	Patients with irritable bowel syndrome	Repetitive transcranial magnetic stimulation	Abdominal pain symptoms are relieved in patients with irritable bowel syndrome and rectal volume pain thresholds are increased in patients with rectal hypersensitivity
Algladi et al. (45)	Patients with irritable bowel syndrome	High frequency repetitive transcranial magnetic stimulation	Increased anal pain threshold and rectal pain threshold
Li et al. (46)	Patients with refractory irritable bowel syndrome and depression	Repetitive transcranial magnetic stimulation	Improved pain, mood, and quality of life

## 5. Conclusion and future perspectives

As one of the common diseases of the gastrointestinal tract, the specific pathogenesis of FBD has not been clarified, which brings a heavy burden to patients physically, mentally, and economically. In recent years, with the development of neurogastroenterology, the brain-gut axis has gradually come into view and has attracted the widespread attention of researchers, and more and more studies have shown that the brain-gut axis may be the pathophysiological alteration of functional enteropathy. As a non-invasive, painless, safe, and reliable new technology, TMS can be used both for the detection of brain function and the localization of the cerebral cortex, providing new ideas for people to study the mechanism of disease occurrence and pathophysiology, and as an emerging treatment modality, providing new means for clinical treatment. In just a few decades, TMS has achieved remarkable results in clinical practice. TMS has been widely used in the treatment of psychiatric disorders and pain relief in China and abroad, but few scholars have studied its application to gastrointestinal disorders. Although only a few researchers in China and abroad have used TMS for the treatment of FBD, patients have shown significant improvement after a period of treatment with no associated adverse effects. Future research efforts on TMS for FBD on a larger scale are needed to explore the underlying mechanisms in greater depth. It is believed that with the continuous development of TMS technology and the progress of clinical medical research, TMS will be better, more closely, and more widely integrated with clinical treatment, providing more possibilities for clinical treatment plans.

## Author contributions

GL and BJ reviewed the relevant literature and wrote the manuscript. ZF designed the structure and revised the manuscript. All authors contributed to the article and approved the submitted version.

## Funding

This work was supported by the National Natural Science Foundation of China (81701965); Natural Science Foundation of Liaoning Province (20180550116 and 2019-MS-069).

## Conflict of interest

The authors declare that the research was conducted in the absence of any commercial or financial relationships that could be construed as a potential conflict of interest.

## Publisher’s note

All claims expressed in this article are solely those of the authors and do not necessarily represent those of their affiliated organizations, or those of the publisher, the editors and the reviewers. Any product that may be evaluated in this article, or claim that may be made by its manufacturer, is not guaranteed or endorsed by the publisher.
